# A System Dynamics Model for Urban Residential Building Stock towards Sustainability: The Case of Jinan, China

**DOI:** 10.3390/ijerph18189520

**Published:** 2021-09-09

**Authors:** Dong Yang, Mengyuan Dang, Lingwen Sun, Feng Han, Feng Shi, Hongbo Zhang, Hongjun Zhang

**Affiliations:** 1 Institute of Science and Technology for Development of Shandong, Qilu University of Technology (Shandong Academy of Sciences), Jinan 250100, China; mengyuan_dang@163.com (M.D.); sunlw@qlu.edu.cn (L.S.); hanf@sdas.org (F.H.); shifeng1224cn@126.com (F.S.); hongbo@zhanlue.net (H.Z.); 2 Energy Research Institute of Shandong Academy of Sciences, Qilu University of Technology (Shandong Academy of Sciences), Jinan 250100, China; zhanghb@qlu.edu.cn

**Keywords:** system dynamics, urban residential building stock, policy measures evaluation, C&D waste management

## Abstract

Resource and environmental issues related to urban building systems have recently become a hot research topic in the field of urban environmental management research. Taking Jinan city as an example, this paper establishes a system dynamic model for an urban residential building stock system. The simulated results show that the urban residential building stock will be 1.99 × 10^8^ m^2^ in 2050; and the annual total demolition buildings will be at 3.36 × 10^6^ m^2^ in 2082. Policy measures were developed based on four important action fields such as per capita floor area (PCFA), the building structure proportion of new construction, lifetime of the residential building, and the recycling of the C&D waste. Among these approaches, the set of policy measures focusing on the recycling of C&D waste appears to be more effective in reducing environmental and resource impacts than the other three fields. It is also found that the recycling of brick and concrete waste plays a considerable role in reducing environment and resource impacts due to the development of urban residential building stock with the lapse of time.

## 1. Introduction

The accumulation and renewal of building stock are the typical characteristic and inevitable result of urbanization. In recent years, with the rapid development of China’s economy, China’s urbanization and urban renewal are also accelerating, including huge construction, renovation, and demolition, which also led to serious excessive consumption of building materials and a sharp increase in construction and demolition waste (C&D waste) [1,2]. On the one hand, construction material such as cement and steel generates many environmental impacts, from extraction, production, and transportation [3]. On the other hand, C&D waste has also changed into one of the biggest environmental problems faced by the urban management department in China. Most of the urban C&D waste is transported to the suburban and rural areas for landfilling. Landfill disposal for C&D waste not only occupies considerable land areas, but also causes many serious environmental problems such as soil and water pollution derived from leachate and greenhouse gas emissions [4]. According to the statistics report from China [5], the yearly output of C&D waste for China is around 1.8 billion tons in 2017; however, less than 10% of C&D waste was practically recycled. Even worse, as the urbanization process in China is accelerating, the environmental and resource impacts due to the building stock will also continue to exist. Therefore, the concern about how to mitigate the environmental and resource impacts has become a main topic for urban governments. From the point of urban sustainable management, it is necessary to analyze the urban building metabolism, identify the characteristics of the flow and stock of urban buildings, and develop the effective measures on reducing the environmental and resource impacts. In recent years, there have been many researchers around the world paying attention to building metabolism. At the urban level, many studies have been extensively conducted in Hong Kong [6], Vienna [7], Wakayama [8], Saxony [9], Beijing [10], and Rio de Janeiro [11]. At the national level, studies also were done in the countries like China [12,13], Japan [14], Norway [15,16], and the Netherlands [17]. Nonetheless, almost all of this research focused only on calculating the construction material flow and stock of building systems but ignored the direct and effective measures to cut down the environmental and resource issues due to building stock variations.

On the other hand, there is also much research on how to lower the environmental impacts due to C&D waste. To name a few, Zhao et al. [18] developed a system dynamics model to assess the alternative recycling facilities under different policy scenarios by comparing their economic viability with the cost-saving ratio of C&D waste management. Bovea and Powell [19] used the LCA method to evaluate the environmental performance of C&D waste. Ding et al. [2] simulated and evaluated the reduction management measures for C&D waste for the design and construction processes based on the system dynamics method. At the same time, there are some ongoing strategies or policies targeting the environmental and resource impacts reduction of urban building stocks. Although most of these studies contemplated specific technologies and related emissions reduction strategies, they ignored the source of environmental and resource impacts and the long-term results of such technologies or measures on reducing the impacts.

Residential buildings are the most important and the fastest-growing type of urban building stock. Therefore, a system dynamics model is performed for urban residential building stock in this paper in order to simulate the dynamic feature of flow and stock and develop a long-term effective measure to reduce the environmental and resource impacts. Besides this, it is also helpful for the policy-makers to evaluate the effective measures for developing urban management on urban residential building stock.

According to the data available, the city of Jinan is chosen as the case study in this research. Jinan is the political, economic, and cultural capital of Shandong province of China. In 2015, the urban population of Jinan was 3.74 million. Just like other metropolises in China, the amount of urban residential buildings constructed in the period from 1978 to 2015 was appeared by a rapid growth in urban population in the city of Jinan. In the meantime, a lot of resources and environmental impacts also appear subsequently. It is estimated that the output of C&D waste in Jinan is about 30–40 million m^3^ per year in this period, and almost all the C&D waste would be landfilled except for steel scraps. Nowadays, there are 12 different landfill sites for C&D waste around this city, and all of them will run out of capacity in 2020 based on the disposal rate at present. Unfortunately, the fact that there is seemingly no appropriate place for the new landfill site in Jinan is exacerbating this situation. Therefore, for the Jinan government, it is becoming one of the important tasks at present and to analyze and solve the environmental problems caused by the extremely urban building stocks. Accordingly, the main objectives of this paper are as follows:

- Develop a system dynamics model for urban residential building stock;
- Simulate and identify the feature of urban residential building stock and relative flow in future;
- Predict the environmental and resource impacts related to the urban residential building stock in future;
- Based on the analysis results, some feasible long-term measures to reduce the environmental and resource problems are proposed and quantitatively evaluated in order to select the most appropriate and effective policy measures to help the decision-making for the urban government.

## 2. Research Methods

The system dynamics (SD) model is a method used to simulate a real time-varying behavior of the complex system with the purpose of simulating the real system and analyzing the important variables in this system based on the principle of system thinking and feedback control theory [20]. According to the research of Sterman [21], the SD modeling method is also carried out in four main phases: (1) Problems and goal identification, (2) SD model building, (3) SD model validation, and (4) Scenario design and policy evaluation. An urban residential buildings system is a complex social-economic system which includes many factors (such as population, PCFA, building structure, building lifetime, construction material demand and consumption, waste, recycling, landfill, etc.) and different stakeholders (citizens, urban manager, construction industry, and the recycling industry). SD provides an effective way of understanding the associated mechanisms and relationships of different system factors and stakeholders. The SD model is established in this paper to simulate and evaluate the development of the urban residential building stock and support a strategy for reducing the environmental and resource impacts for urban sustainable development. In addition, the software Vensim Version 7.0 (company, Ventana Systems Inc, Harvard, MA, USA) and Stella Architect Version 1.9 (Isee Systems, Lebanon, USA) are applied to construct the SD model.

### 2.1. Causal Loop Diagram

**Figure 1:**
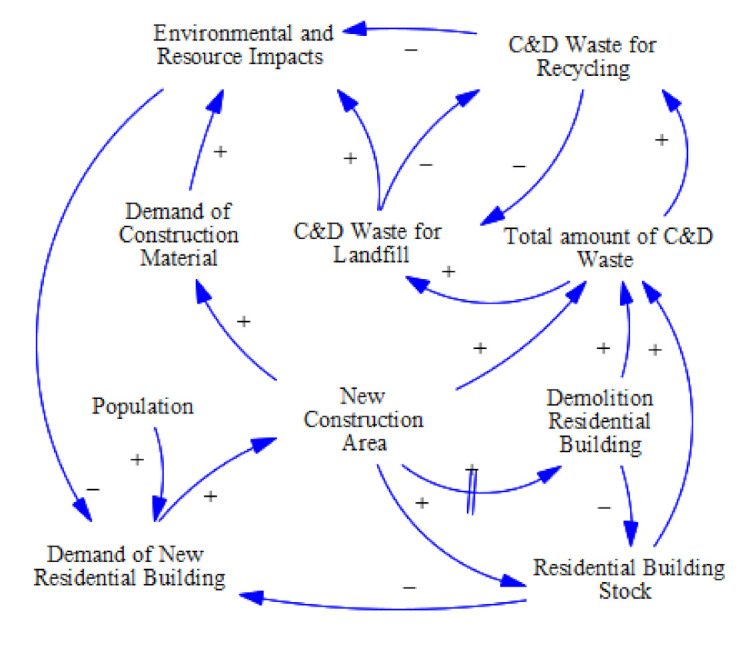
Causal loop diagram of the urban residential building stock.

A causal loop diagram (CLD) for urban residential building system is shown in Figure 1 based on the Vensim Version 7.0. It is clearly seen that there are several causal loops in this CLD, and the main feed loops are provided as follows:

1. Demand of new residential building—(+) New construction area—(+) Residential building stock—(−) Demand of new residential building;
2. Demand of new residential building—(+) New construction area—(+) Demolition residential building—(−) Residential building stock—(+) Demand of new residential building;
3. Demand of new residential building—(+) New construction area—(+) Total amount of C&D waste—(+) C&D waste for landfill—(+) Environmental and resource impacts—(−) Demand of new residential building;
4. Demand of new residential building—(+) New construction area—(+) Demolition residential building—(+) Total amount of C&D waste—(+) C&D waste for recycling—(−) Environmental and resource impacts—(+) Demand of new residential building;
5. Demand of new residential building—(+) New construction area—(+) Residential building stock—(+) Total amount of C&D waste—(+) C&D waste for recycling—(−) Environmental and resource impacts—(+) Demand of new residential building;
6. C&D waste for reusing and recycling—(−) C&D waste for landfill—(−) C&D waste for recycling;
7. Demand of new residential building—(+) New construction area—(+) Demand of construction material—(+) Environmental and resource impacts—(−) Demand of new residential building.

For example, in the feed loop (1), if the urban population increases, the demand for new residential building increases, which would result in an increase in residential building stock and future demolition residential buildings. This can further reduce the demand for new residential building stock. Meanwhile, the feed loop (6) is typical negative feedback that has two opposite parameters: C&D waste disposal by landfill or recycling. Based on different disposal methods, the C&D waste is divided into C&D waste for landfill and for recycling. Increasing the number of C&D for landfills would result in the number of C&D for recycling decrease, and vice versa.

### 2.2. Data Collection

Data of the different parameters are the vital component which directly determines the accuracy of the SD model. For this study, the data are collected from the governmental reports, papers, books, and some related websites. Some information about the key variables in the model such as units, values, and data sources is presented in Table 1. Some variables may not be available through existing statistics data, so the acquisition of this data can only be conducted through system analysis or table functions of SD theory.

### 2.3. Stock and Flow Diagram

**Figure 2:**
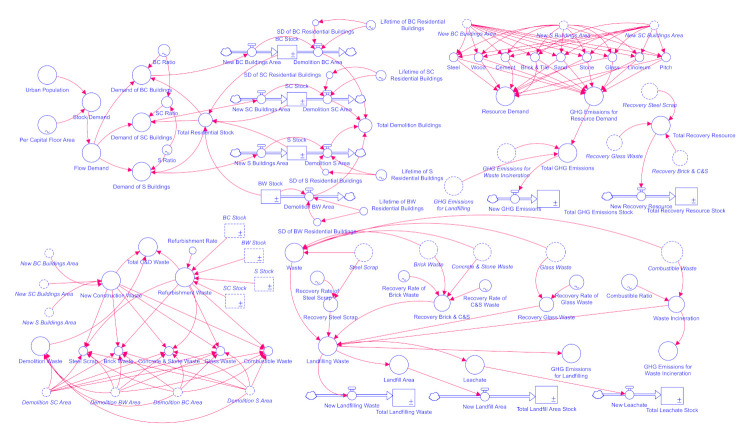
Stock and flow diagram of urban residential building stock.

The stock and flow diagram for urban residential building stock is shown in Figure 2 based on the SD theory by Stella Architect Version 1.9 in order to analyze the dynamic features of urban residential building stock and relative flow, simulate the environmental and resource impact, as well as propose and evaluate several measures for mitigating the impacts for Jinan city in the future. The key parameters of the model are given for more details and the mathematical relationship between the parameters are also shown in the following section.

1. Urban population

Urban population is a major parameter and driving force for urban residential building stock. As shown before, the urban population of Jinan grew rapidly from 1978 to 2015 as a consequence of economic development and rapid urbanization. The urban population of Jinan was 3.74 million in 2015, with a 1.7% increase per year from 1978. The urban population data from 1978–2015 can be obtained from the Jinan statistics yearbooks (1978–2016). Based on the logistics population model [22], the urban population logistics model of Jinan (Equation (1)) is set up using the data of the urban population from 1978 to 2015. By this equation, we can acquire the urban population data from 2016 to 2100:

(1) $$P\left(t\right)=10,000*\frac{401.9}{1+e^{-\left(0.07623*t-151.1\right)}}\;$$

$P\left(t\right)$ is the urban population of the case city in year *t*, Unit: per person; *t* is the current year.

2. Per capita floor area

Per capita floor area (PCFA) is also a crucial factor for estimating the urban residential building stock which represents the quality of life of urban citizens to some degree. The PCFA data of Jinan from 1978 to 2015 were obtained from the Jinan Statistical Yearbook. The PCFA of Jinan is 45 m^2^ in 2015. According to the research of Shi [12] and Huang [13], we assumed that the future PCFA for Jinan will reach 50 m^2^ in 2050 and remain the same from 2050 to 2100.

3. Building structures of new constructions of residential building stock

There are four main building structures in the urban residential building stock system of Jinan: Brick-wood building (BW), Brick-concrete building (BC), Steel-concrete building (SC), and Steel building (S). According to the survey, the BC and SC buildings are the mainstream building structures in the new construction. Since almost no BW building is constructed in Jinan from 2000, the proportion of BW of new construction is assumed to be 0 in the model. The S building indicated the development trend of the residential building. We got the data of the proportion of different building types of Jinan on the basis of the Jinan statistics census [23,24]. Based on the table function from SD theory, we can determine the proportion of new constructions of residential building stock with different building structures from 1978 to 2015.

4. Urban residential building stock

The urban residential building stock is the accumulation of residential building area per year defined as a stock and estimated based on the urban population and PCFA of Jinan each year (Equation (2)):

(2) $$S\left(t\right)=P\left(t\right)\times PCFA\left(t\right)$$

(3) $$S\left(t\right)=S_{BW}\left(t\right)+S_{BC}\left(t\right)+S_{SC}\left(t\right)+S_{S}\left(t\right)$$

where *t* is the current year; $S\left(t\right)$ is urban residential building stock in year *t*; $P\left(t\right)$ is urban population;$\;PCFA\left(t\right)$ is per capita floor area of urban residential building in year *t*;$\;S_{BW}\left(t\right)$ is urban residential building stock with BW building structure of year *t*; $S_{BC}\left(t\right)$ is urban residential building stock with BC building structure of year *t*;$\;S_{SC}\left(t\right)$ is urban residential building stock with SC building structure of year *t*;$\;S_{S}\left(t\right)$ is urban residential building stock with S building structure in year *t*.

5. New urban residential building construction

The new urban residential building construction is defined as a flow, which is basically the number of new residential building constructions for the new demand of urban residential building stock due to urban population growth and old building demolition. The data of the new residential building construction per year are calculated through the equation below:

(4) $$F_{in}\left(t\right)=S\left(t\right)-S\left(t-1\right)+F_{out}\left(t\right)$$

(5) $$N_{i}\left(t\right)=F_{in}\left(t\right)\times R_{i}\left(t\right)$$

where *t* is the current year; $S\left(t\right)$ is the urban residential stock of year *t*;$\;S\left(t-1\right)$ is urban residential stock in year *t −* 1; $F_{in}\left(t\right)$ is total new construction of urban residential buildings in year *t*; $F_{out}\left(t\right)$ is total demolished residential buildings in year *t*; $N_{i}\left(t\right)$ is new constructions of urban residential building with building structure *i* in year *t*;$\;R_{i}\left(t\right)$ is the ratio of new construction urban residential building with building structure *i* in year *t*.

6. Demolished residential building area

Demolished residential building area is also a significant flow variable related to the new residential building construction. It is basically the number of demolished residential building area due to the end of the old building lifetime and urban renewal practice. This variable directly determines the output of the C&D waste. 

Owing to the absence of accurate statistic data, the demolished residential building area with different building structure is alternatively calculated on the basis of the previous research [10,25] as the equations below:

(6) $$F_{out}\left(t\right)=\sum_{i}D_{i}\left(t\right)\;$$

(7) $$D_{i}\left(t\right)=\sum_{t_{0}}^{t^{\prime }}L_{i}\left(t,t^{\prime }\right)\times N_{i}\left(t^{\prime }\right)$$

(8) $$L\left(t,t^{\prime }\right)=\frac{1}{\sigma \sqrt{2\pi }}e^{\frac{-{\left(t-t^{\prime }-\tau \right)}^{2}}{2\sigma^{2}}}$$

where $t_{0}$ is start year; *t* is current year; $F_{out}\left(t\right)$ is total demolished residential building of the year *t*; $D_{i}\left(t\right)$ is demolished residential building with building structure *I* in year *t*; $L\left(t,t^{\prime }\right)$ is the lifetime distribution of urban residential buildings with building structure *i* of year *t*. It is probable that the building built in year $t^{\prime }$ will be demolished in year t. τ is the lifetime of the residential building and σ presents the standard deviation of the residential building lifetime, σ=0.3τ.

7. Lifetime of the residential building

The lifetime is an indispensable variable in calculating demolished residential building area per year. Generally, different urban residential buildings with different structures also have a different design lifetime. However, the actual service lifetime of residential buildings is much shorter than the design lifetime in Jinan because of unreasonable urban planning and renewal. There are many studies mentioning that the average lifetime of almost all urban residential buildings is approximately 30–40 years [26,27]. Based on the actual situation of Jinan, the lifetime of the residential buildings with different building structures is assumed in Table 2.

8. Environmental and resource impacts

Environmental and resource impacts are defined as the environmental emissions and resource consumption caused by the construction materials’ manufacturing, construction, refurbishment, demolition, and disposal of C&D waste of the residential building stock. There are many environmental issues related to the C&D disposal and the production of construction such as energy and resource consumption, water pollution, and GHG emission. According to some research [28,29,30], five different environment and resource impacts indicators were selected including (1) construction material consumption, (2) C&D waste by landfill, (3) land space occupation by C&D waste landfill; (4) leachate by C&D waste landfill, and (5) GHG emissions due to construction materials, construction, refurbishment, demolition, and disposal of C&D waste. These parameters can be calculated through the equations as follows:

(9) $$W_{R}\left(t\right)=\sum_{j}W_{j}\left(t\right)=\sum_{i,j}N_{i}\left(t\right)\times MI_{i,j}$$

(10) $$M_{l}\left(t\right)=M_{C\&D}\left(t\right)-M_{R}\left(t\right)-M_{I}\left(t\right)$$

(11) $$LO\left(t\right)=M_{l}\left(t\right)\times C_{LO}$$

(12) $$LE\left(t\right)=M_{l}\left(t\right)\times C_{LE}$$

(13) $$GHG\left(t\right)=M_{l}\left(t\right)\times C_{l}+M_{I}\left(t\right)\times C_{I}+\sum_{j}W_{j}\left(t\right)\times C_{j}$$

where $W_{R}\left(t\right)$ is the consumption of construction material for new residential buildings in year *t*; $W_{j}\left(t\right)$ is the consumption of construction material for new residential building with building structure *j* in year *t*; $N_{i}\left(t\right)$ is the new construction residential building with building structure *i* in year *t*;$\;MI_{i,j}$ is material intensity of construction material *j* with building structure *i*; $M_{l}\left(t\right)$ is mass of C&D waste disposal by landfill in year *t*; $M_{C\&D}\left(t\right)$ is mass of total C&D waste in year *t*; $M_{R}\left(t\right)$ is mass of C&D waste for recovering in year *t*;$\;M_{I}\left(t\right)$ is mass of C&D waste for incineration in year *t*; $LO\left(t\right)$ is the area of the land space occupation by C&D waste landfill of year *t*; $C_{LO}$ is the coefficient of land space occupation; $LE\left(t\right)$ is the volume of the leachate due to the C&D waste landfill;$\;C_{LE}$ is coefficient of leachate; $GHG\left(t\right)$ is the total GHG emission in year *t*; $C_{j}$ is the GHG emission coefficient of the construction material *j*;$\;C_{l}$ is the GHG emission coefficient of C&D waste disposal by landfill;$\;C_{I}$ is the GHG emission coefficient with C&D waste for incineration.

The material intensity of the residential building with different building structure data is exported from the research of Shi [12], while the main coefficients for environmental and resource impacts are taken from the Ecoinvent database [31] and related publications shown in Table 3.

### 2.4. SD Model Validation

Model validation is an important and necessary step of SD modeling, which is represented by the fact that the accuracy of the model can be judged to some extent. The comparative validation method is often used for model validation [32]. In order to verify whether the simulation results were consistent with the actual situation, this study performed the comparative verification test of the SD method. Due to lack of the statistical data of C&D waste in Jinan during 1978 to 2015, verification of the model is performed by comparing the estimates produced by the model with actual historical data using two key indicators: (1) urban population of Jinan and (2) urban residential building stock. The error between the actual data and simulation data was estimated by Equation (14):

(14) $$\mathrm{Error}=\frac{\left|\tilde{s}-\tilde{a}\right|}{\tilde{a}}$$

$\tilde{a}$ represents the average value of actual data;$\;\tilde{s}$ represents the average value of simulated data.

Based on Barlas [33], if the accuracy of the error is less than 5%, it means that the SD model could be effectively verified by actual data. The error of the two parameters is presented in Table 4. It is obvious from this table that the model simulation is compatible with the actual data. This also indicates that the model established in this paper is accurate and successful and can be applicable for the research aim simulating and evaluating the urban residential building stock and relative resource and environment impacts.

## 3. Simulation Results

**Figure 3:**
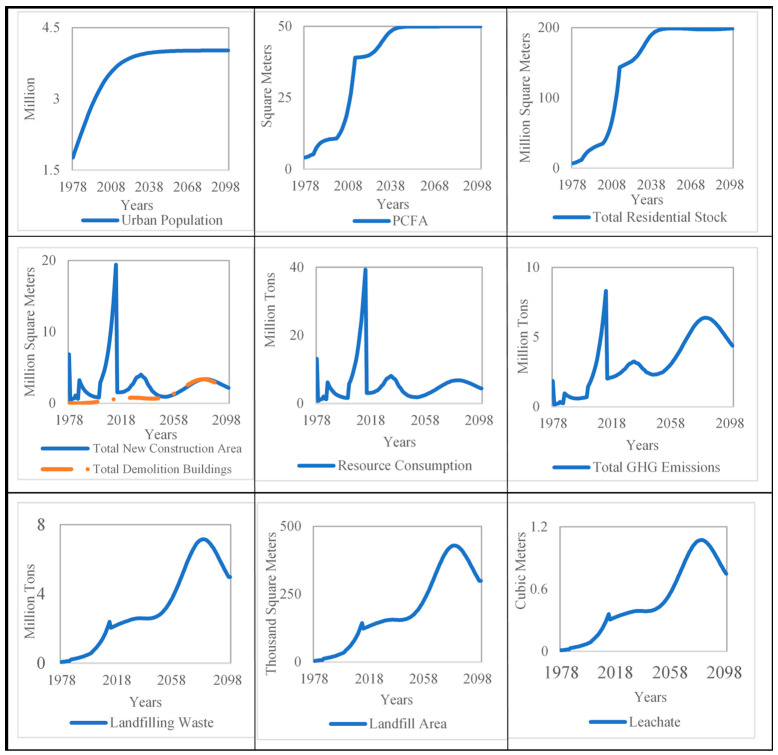
Simulation results of urban residential building stock of Jinan from 1978 to 2100.

Figure 3 shows the simulation results of urban residential building stock development in Jinan from 1978 to 2100. It can be seen that the urban building stock of Jinan increased rapidly from 1978 correspondent to the dramatic increase in urban population and PCFA. The urban population of Jinan will be 4.02 × 10^6^ person in 2100, while the urban residential building stock will be at 1.99 × 10^8^ m^2^ in 2050, and will maintain a slight fluctuation from 2050 to 2100.

The result also illustrates the trend of total new construction area and total demolition buildings of Jinan during the simulation periods from Figure 3. It can be seen that the new construction area periodically fluctuates from 2015 to 2100. The new construction area will reach the first peak at 4.03 × 10^6^ m^2^ in 2033, and the second peak at 3.38 × 10^6^ m^2^ in 2082, respectively. The total demolition of buildings of Jinan increases slowly between 2015 and 2050, showing almost the same trend compared to the total new construction area during 2050 and 2100, and also will be a peak at 3.36 × 10^6^ m^2^ in 2082. This change also indicates that the massive residential buildings construction from 2001 to 2015 would be at the end of the lifetime from 2050 to 2080.

The change of main environmental and resource impacts caused by an increase in urban residential during the simulation periods also were presented in Figure 3. It can be seen that the environmental problems on the C&D waste for landfill (C&D waste, land occupation, and leachate) have a similar tendency during the simulation periods. They are all increased from 2015 to 2082, when all of them first increase from 2015 to 2082 and then reach a peak in 2082 before declining until 2100. Namely, the output of C&D waste for landfill per year will be at a peak of 7.16 × 10^6^ ton in 2082, and, based on the current status of the C&D disposal in Jinan, the huge C&D waste produced in the future will seriously influence the sustainability management of Jinan. Thereby, the GHG emissions and construction material consumption trends have positive relevance with the new construction area per year during the simulation periods. The GHG emissions and resource consumption per year will reach the first peak at 3.25 × 10^6^ tons CO_2_eq and 8.16 × 10^6^ tons in 2033, and the second peak at 6.39 × 10^6^ ton CO_2_eq and 6.84 × 10^6^ tons in 2082, respectively.

## 4. Scenario Analysis

### 4.1. Policy Scenario Set

There are four main fields were taken into account on the policy scenario set such as PCFA, the building structure proportion of new construction, lifetime of the residential building, and the recycling of the C&D waste. This is mainly because the amount of C&D waste and construction materials consumption associated with urban residential building stock are directly or indirectly affected by the above four fields. 

Based on these fields, combined with the characteristics of urban management, several policy measures have been proposed to reduce the resource and environmental impacts due to urban residential building stock in the future. At the same time, a series of complex policy measures (combination of different single policies) were proposed in order to analyze and ensure the effectiveness of the measures. More information on the above individual measures and comprehensive measures is shown in Table 5.

The base case is the scenario without any measures. After the base case is defined, four main single policy implications are considered.

1. Policy 1

According to the research of Bai et al. [34], the average PCFA of Chinese urban residential areas will reach a peak at 45 m^2^ in the future. Moreover, the further increase in PCFA does not mean an increase in living standards. Rather, this indicates a waste of building resources. In 2015, PCFA of Jinan urban residential building stood at 45 m^2^, and it is likely to increase toward the future. Therefore, we set policy 1 as a compact PCFA policy, that is, by controlling the size of the house type and reducing the speed of land transfer from urban government, the PCFA of Jinan urban is restricted at 45 m^2^ without any further growth. Policy 1 aims to reduce the new construction area of residential buildings by controlling the PCFA.

2. Policy 2

Compared with steel-concrete, brick-concrete structures and steel-structured buildings are considered to be a new generation of energy-efficient buildings that need to be promoted due to their strong structure, low resource consumption, and long service life. According to Shandong’s thirteenth five-year circular economy plan [35], the Jinan government has requested to vigorously promote the increase in the proportion of steel structures in new buildings. On this basis, we have formulated Policy 2 as a new technology policy. The proportion of steel structures in Jinan’s new residential buildings is about 5% in 2015. Based on the building level of developed countries, we set the policy 2 that the proportion of steel structures in new urban residential buildings in Jinan will reach 65% in 2050. Policy 2 aims to reduce the demolition area per year by increasing the proportion of the new construction rate of the residential building with steel structures.

3. Policy 3

As mentioned earlier, the lifetime of residential building is extremely important to the residential building flow and related environmental and resource issues. Many researchers show that prolonging the lifetime of urban residential buildings will be beneficial to reduce the environmental and resource impacts of urban building system by avoiding unreasonable urban planning and demolition [36,37]. According to the documents of the Ministry of Housing and urban-rural development of China [38], cities around China should actively extend the service lifetime of various buildings during urban renewal to reduce the resource and environmental impacts. On this basis, we have developed policy 3 as the long lifetime for residential building policy that extends the life of buildings by implementing scientific planning and improving the quality of buildings based on existing short lifetime buildings. Policy 3 aims to reduce the new construction and demolition area by prolonging the lifetime of residential buildings.

4. Policy 4

Increasing the utilization rate of construction waste resources can effectively reduce the solid waste generated by urban demolition. At present, the recycling rate of C&D waste in Jinan is very low. In addition to steel scrap, most C&D waste are landfilled for disposal. In order to solve this problem, Regulations on Urban Construction Waste Management in Jinan [39] are approved by the Jinan government, requiring the recycling of urban construction waste. From this strategy, we have established policy 4 as a high recycle rate policy, through the recovery of steel, glass, and various waste bricks mixed concrete materials, and thereby it is possible to achieve better recycling for C&D waste by 2050. Policy 4 aims to reduce the environmental and resource issues by increasing the recycle ratio of C&D waste. After that, in order to enhance the effect of single measures, another 10 complex policies are generated for simulation and evaluation analysis.

### 4.2. Scenario Analysis Results

#### 4.2.1. Environmental Impacts

**Figure 4:**
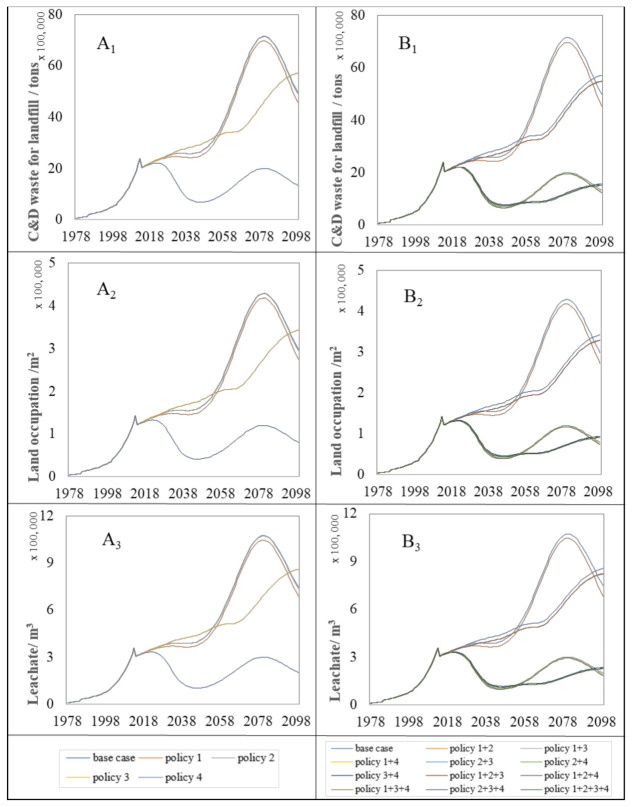
Annual environmental impacts trends for proposed policy measures. A_1_ and B_1_ represent the impact trend of single policy and complex policy on C&D waste for landfill, respectively. A_2_ and B_2_ are the impact trend of single policy and complex policy on land occupation, respectively.A_3_ and B_3_ indicate the impact trend of leachate under single policy and complex policy, respectively.

As mentioned before, there are three main environmental impacts indicators considered in this section: C&D waste for landfill, land occupation, and leachate. Figure 4 illustrates three environmental impacts on the C&D waste for landfill trends of the case and single and complex proposed policy measures.

The base case presents the basic policy scenario, which shows a situation in which none of the measures were implemented. According to Figure 4, these policy measures enhance the reduction in the annual environmental problems on the C&D waste for landfill compared with the base case. The policy measures focusing on improving the C&D waste recycling rate contributes a more considerable effect on the annual environmental problem reduction compared to other single policies. For instance, it can be seen from Figure 4(A_1_–A_3_) that policy 4 is the most effective measures to lower the annual output of C&D waste, land space occupation, and leachate for landfill in the single policies. These three major environmental impacts peak around 2080, when policy 4 reduces environmental impacts by approximately 72% compared to policy 1 and policy 2. Meanwhile, the complex policy measures including policy 4, in particular policy 2+3+4 and policy 1+2+3+4, will be more effective than the others to weaken these environmental impacts from 2016 to 2100 (Figure 4(B_1_–B_3_)). Apart from that, the policy measures focusing on prolonging the lifetime of residential buildings also proved to be helpful to reduce the annual environmental problems on the C&D waste for landfill. Conversely, policy 2 focusing on increasing the steel structure is conceivably the least effective approach to reduce the annual environmental problems in all policy measures proposed.

In addition to annual environmental problems, it is also crucial to evaluate the effect of proposed measures to the accumulation of environmental impacts (environmental impacts stock). Compared with the annual environmental impacts, the long-term environmental impacts stocks of the C&D waste landfilled in the land should be given more attention. Proposed policy measures are quantitatively evaluated on the C&D waste for landfill based on their effect of the environmental impacts within the period of 2016 to 2100.

**Figure 5:**
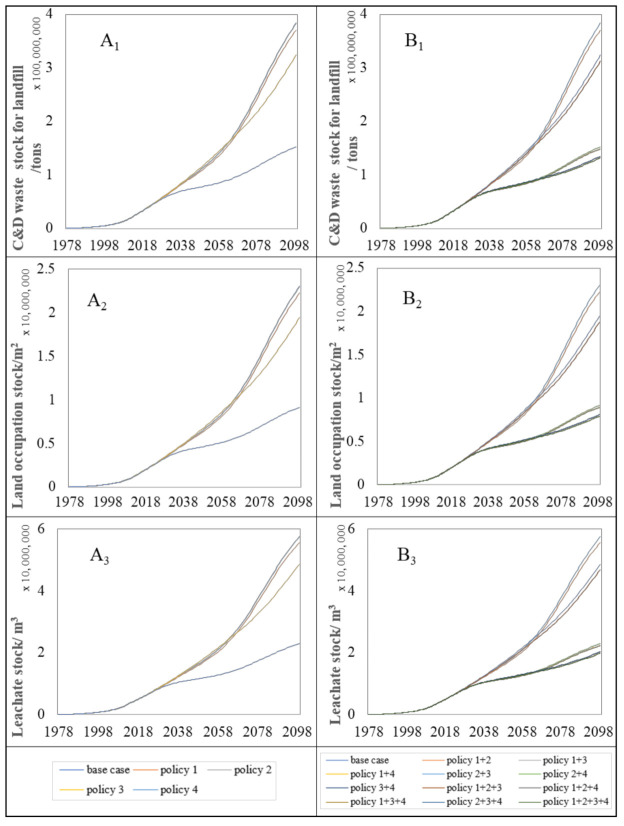
Trend of environmental impacts stock for proposed policy measures. A_1_ and B_1_ represent the trend associated with environmental impacts stock of C&D waste for landfill under single policy and complex policy, respectively. A_2_ and B_2_ are the trend associated with environmental impacts stock of land occupation under single policy and complex policy, respectively. A_3_ and B_3_ indicate the trend associated with environmental impacts stock of leachate under single policy and complex policy, respectively.

Figure 5 shows the trends associated with environmental impacts stock on the C&D waste for landfill as a result of policy implementation. According to this figure, policy 4 was also found to have the greatest action on environmental impact reduction in the single policy. Despite the complex policy measures, none of them can help with slowing down the increasing trends until 2100 without using policy 4.

In addition to the changes in trends of annual and environment impacts stock on the C&D waste for landfill, it is critical to summarize the reduction percentage made by each policy from 2016 to 2100. 

**Figure 6:**
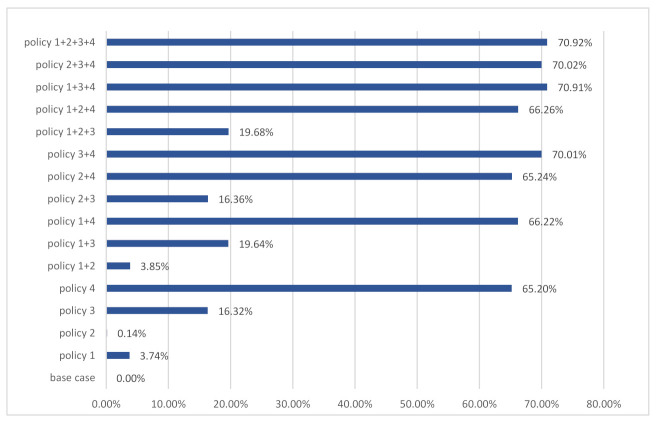
The effect of policy measures on the environmental impacts stocks until 2100.

Based on an analysis of the annual accumulation of environmental impact trends of C&D waste disposal by landfill, the effect of each policy on environmental impact reductions from 2016 to 2100 is also summarized. The effects of the proposed policy measures on environmental impact stocks (percentage reduction) were shown in Figure 6. Among the proposed policy measures, the set of policies that emphasize increasing the recycling rate of the C&D waste are found to be much more effective than the others, followed by the policies focused on the lifetime of residential buildings.

On the other hand, the policy measures targeting the PCFA and the building structure in the new construction area are slightly ineffective and inefficient toward environmental problems stock reduction. The highest reduction of complex and single policy measures was achieved by the policy 1+2+3+4 and policy 4 with 70.92% and 65.02%, respectively.

#### 4.2.2. Resource Impacts

**Figure 7:**
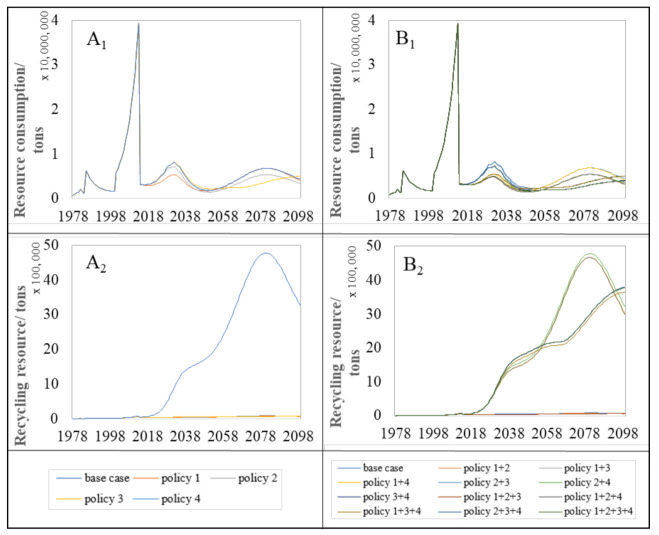
Annual resource impacts trends for base case and proposed policy measures. A_1_ and B_1_ represent the impact trend of single policy and complex policy on resource consumption, respectively. A_2_ and B_2_ are the impact trend of single policy and complex policy on recycling resource, respectively.

Compared with the base case, all policy measures enhance the decrease in the annual resource consumption trends except policy 4 based on Figure 7(A_1_,B_1_). The reason is that the aim of policy 4 did not directly reduce the construction material consumption. Rather, it can increase the recycle rate of resources such as steel scraps, glass, and brick waste from the C&D waste. As shown in Figure 7(A_2_), the amount of recycling resource by policy 4 in 2081 is about 62 times that of other policies. Unlike the environment impacts on the C&D waste for landfill, policies 1, 2, and 3 are all efficient frameworks in the single means to reduce the construction resource consumption per year because of the rapid growth of urban residential building stock in Jinan. Among these, policy 3 has the highest potential to reduce the annual resource consumption in the single policy measure (Figure 7(A_1_)). Overall, the sum of the policy 1+2+3+4 and policy 1+2+3 that combines the different single plans will constitute the most powerful measures to reduce the consumption of construction resources in the future (Figure 7(B_1_)). 

**Figure 8:**
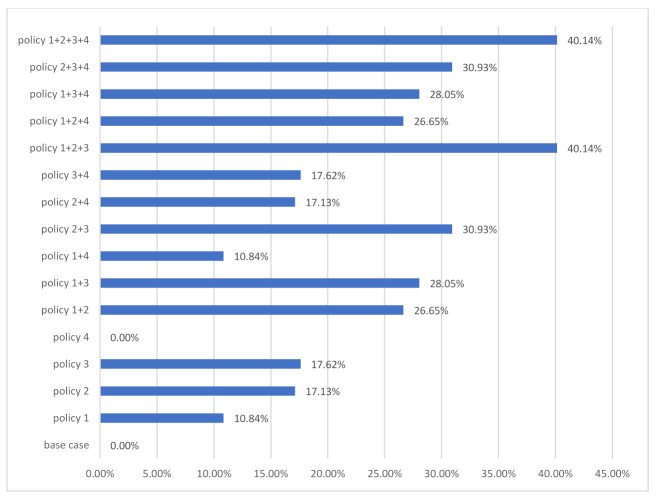
The effect of policy measures on resource consumption by policy measures until 2100.

The effect of the proposed policy measures on building resource consumption from 2016 to 2100 was summarized and presented in Figure 8. It is clearly found that the highest reductions of the complex policy measures were achieved by the policy 1+2+3+4 and policy 1+2+3 with 40.14%, respectively. 

To reduce resource consumption due to the residential building stock, it is necessary to improve the recycling rate of the source from C&D waste such as steel scrap, glass waste, brick, and concrete waste. Therefore, the recycling of C&D is also considered in this section.

As shown in Figure 7(A_2_,B_2_), it is obvious that only policy 4 can increase the recycle resource from the C&D waste in the single policy compared with the base case. The complex policy measures which include policy 4 can better strengthen the effectiveness of increasing the recycle resources from the C&D waste in comparison with other complex policy measures.

#### 4.2.3. GHG Emission

**Figure 9:**
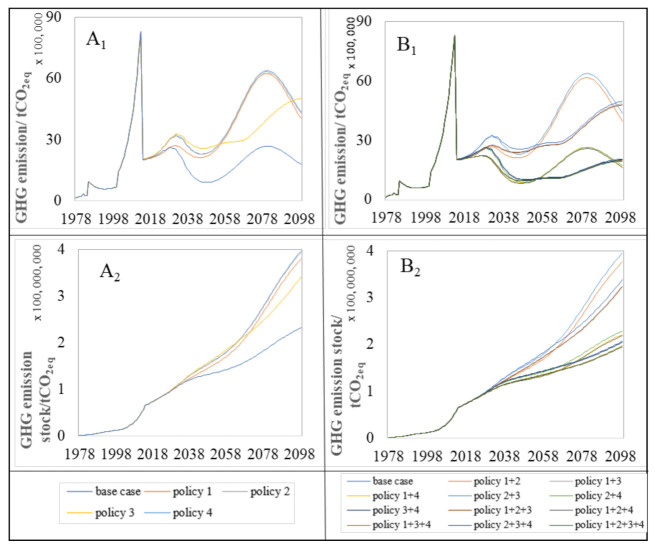
Trend of annual and stock of GHG emission for base case and proposed policy measures. A_1_ and B_1_ represent the impact trend of single policy and complex policy on GHG emission, respectively. A_2_ and B_2_ are the impact trend of single policy and complex policy on GHG emission stock, respectively.

Figure 9 illustrates the trends of the annual GHG emissions and GHG emissions stock under the base case and other different policy scenarios.

According to Figure 9(A_1_,B_1_), all of the policy measures enhance the reduction in the annual GHG emission as opposed to the base case. Similar to the previous environmental and resource impacts, the policy measures focusing on the increase in the recycling rate of the C&D waste create better potential in mitigating annual GHG emission compared to other policy measures. For instance, policy 4 is the most effective policy measures to reduce the annual GHG emission in the single policy measures, which is followed by the policy 3. In contrast, policy 2 was regarded as the least effective solution to reduce the annual GHG emissions in the single policy measures. It is shown that the disposal of C&D waste will lead to an increasing amount of the annual GHG emissions with the slow growth of the residential building.

The long-term effect of the GHG emission stock stored in the atmosphere should also be analyzed, and the proposed policy measures are quantitatively evaluated based on their reduction effect of the GHG emission within the period of 2016 to 2100. Figure 9(A_2_,B_2_) show the trends associated with GHG emission stock as a result of the proposal policy measures’ action plans. 

From Figure 9(A_2_,B_2_), we can see the trends of GHG emissions stocks under the base case and different proposed policy measures. It is found that policy 4 seemingly performed the greatest mitigation action on the GHG emission stock in the single policy measures; however, it did not help with slowing down the increasing trend by 2100. Instead, the policy 1+2+3+4 appears to be the best policy measure to halt the increasing trend in the GHG emission stock.

**Figure 10:**
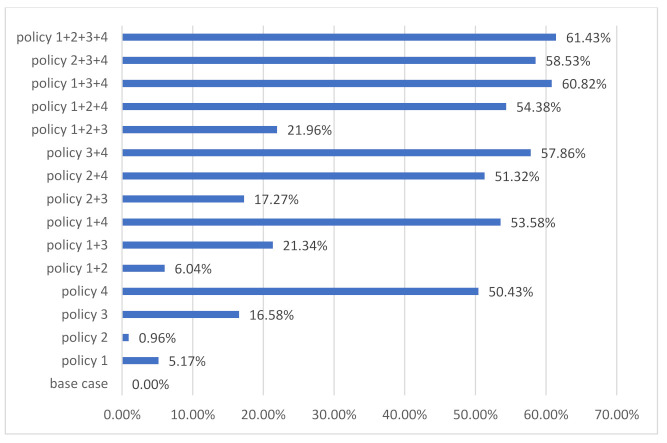
The effect of policy measures on GHG emission stock by policy measures’ implications until 2100.

In addition to the annual GHG emission trend, it is necessary to evaluate the effect of the proposed policy measures from 2016 to 2100. The results in the GHG emission stock reduction by the proposed policy measures are shown in Figure 10.

As illustrated from this figure, the highest reduction of the single and complex policy measures was achieved by policy 4 and policy 1+2+3+4 with 50.43% and 61.43%, respectively.

Through the above analysis, it is obvious that improving the recycling rate of C&D waste is one of the most effective approaches to reduce the resource and environmental problems brought by the development of residential buildings stock. Therefore, this should also become the core emphasis of the urban residential building stock management for government. At present, the policy and law on the C&D waste management and recycling are relatively lacking in China. Most of the C&D waste is disposed of by landfilling. Thus, it is necessary for the Jinan government to draw on advanced experience from developed countries such as Japan and Germany, combine the actual feature of Jinan, and actively formulate the regulations on improving the recycling of C&D waste. By promoting C&D waste recycling technologies, establishing standards for recycled products, financial subsidies, tax incentives and other measures, it is crucial to improve the utilization rate of C&D waste and achieve the high recycling rate of C&D waste in order to reduce the environmental and resource impacts due to the urban residential building stocks by 2050.

### 4.3. Sensitivity Analysis

As shown before, policy 4 that focuses on the recycling rate of the C&D waste is the best single policy measures to lower the environmental and resource impacts due to the urban residential building stock development. The main factors dominating the recycling of the C&D waste in the SD model can be identified as follows: (1) the recycle rate of steel scrap; (2) the recycle rate of brick and concrete waste; (3) the recycle rate of glass waste; and (4) combustible rate of combustible waste. In order to estimate the effect of the four parameters on the environmental and resource impacts, sensitivity analysis is performed in this SD model. C&D waste stock for landfill and GHG emission stock are taken as examples. For variables on the recycling rate of steel scrap, sensitivity analysis (R1, R2, R3, R4) represents four results based on different recycling rate of steel scrap (0.6, 0.7, 0.8, 0.9), respectively. For the other three parameters, sensitivity analysis (R1, R2, R3, R4) represents four results based on different parameters (0, 0.1, 0.2, 0.3), respectively. 

**Figure 11:**
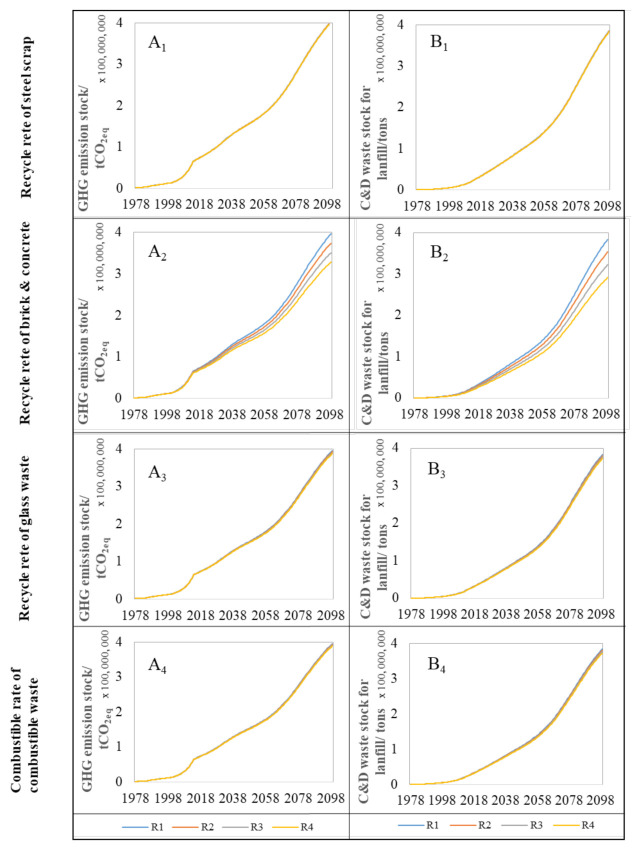
The simulation of the sensitive analysis. A_1_, A_2_, A_3_ and A_4_ represent the impacts of different recycle rate of scrap steel, different recycle rate of brick & concrete, different recycle rate of glass waste and different combustible rate of combustible waste on GHG emission stock. B_1_, B_2_, B_3_ and B_4_ represent the impacts of different recycle rate of scrap steel, different recycle rate of brick & concrete, different recycle rate of glass waste and different combustible rate of combustible waste on C&D waste stock for landfill.

Figure 11 shows that the recycling rate of steel scrap, the recycling rate of glass waste, and the combustible rate of combustible waste have almost no influence on the C&D waste stock for landfill and GHG emission stock, since the proportion of the steel scrap, glass waste, and combustion waste in the C&D waste was remarkably low. An increase in the recycle rate of steel scrap, the recycle rate of glass waste, and a combustible ratio of 10% leads to the decrease of the C&D waste stock for landfill of 0.18%, 0.93%, and 1.01% in 2100, and decrease the GHG emission stock of 0.13%, 0.66%, and 0.53%, respectively. 

Through the above analysis, it is obvious that improving the recycling rate of C&D waste is one of the most effective approaches to reduce the resource and environmental problems brought by the development of residential buildings stock. Therefore, this should also become the core emphasis of the urban residential building stock management for government. At present, the policy and law on the C&D waste management and recycling are relatively lacking in China. Most of the C&D waste is disposed of by landfilling. Therefore, it is necessary for the Jinan government to draw on advanced experience from developed countries such as Japan and Germany, combine the actual feature of Jinan, and actively formulate the regulations on improving the recycling of C&D waste. By promoting C&D waste recycling technologies, establishing standards for recycled products, financial subsidies, tax incentives, and other measures, it is crucial to improve the utilization rate of C&D waste and achieve the high recycling rate of C&D waste in order to reduce the environmental and resource impacts due to the urban residential building stocks by 2050.

## 5. Conclusions

In this paper, the residential building stock in Jinan is modeled by a system dynamics approach. The simulated results show that the urban residential building stock will peak at 1.99 × 10^8^ m^2^ in 2050, which is 1.35 times the residential building stock in the base year of 2015; and the annual total demolition buildings will be in the peak at 3.36 × 10^6^ m^2^ in 2082, which is 5.30 times the total amount of demolition areas in the base year of 2015. The stock of C&D waste for landfill, land space occupation for landfill, leachate, and the GHG emissions due to the urban building stock from 2016 to 2100 will be at 3.60 × 10^8^ tons, 2.16 × 10^7^ m^2^, 5.40 × 10^7^ m^3^, and 3.31 × 10^8^ tons CO_2_eq, respectively. 

The projections on the environmental and resource impacts are estimated on 14 different policy scenarios from 2015 to 2100. The goal of this research is to select the best policy measure options which can reduce the continuous increasing trend of the environmental and resource impacts derived from the increase in building stock. Effective solutions to mitigate such issues are heavily dependent on the four key factors as follows: (a) PCFA; (b) the building structure proportion of new construction; (c) lifetime of the residential building; and (d) the recycling rate of the C&D waste. Nonetheless, the policies focusing only on decreasing PCFA, modifying the building structure, and prolonging the lifetime of the residential building can never accomplish the environmental and resource impact mitigation in the future, unless the policy of C&D waste recycling enhancement is seriously undertaken. 

From the simulations and related analysis, policy 4 and policy 1+2+3+4 are found to be the most successful in the single and complex policy measures among the 14 different policy scenarios, respectively.

Among the different methods to increase the recycling rate of the C&D waste, the recycling rate of brick and concrete waste has the biggest influence on the environmental and resource impacts. Particularly, an increase in recycling rate of brick and concrete waste plays a progressively important role in reducing environment and resource impacts with regard to the development of urban residential building stock with the lapse of time.

This research would offer policymakers indispensable knowledge to further develop policies with a more essential contribution in order to ensure the substantial reduction in the urban residential building stock and the effective C&D waste management in Jinan City.

## Figures and Tables

**Table 1:** Description of some key variables.

Variable	Description	Unit	Initial Value	Date Source
Urban population	the total number of urban people	Million	181	Jinan statistic yearbook (1984)
PCFA	per capita residential building floor area	m^2^	4.06	Jinan statistic yearbook (1984)
BC ratio	the ratio of new construction area with BC structure	%	74.79	Tabulation on the 2000 population census of Jinan city (2002)
SC ration	the ratio of new construction area with SC structure	%	20.21	Tabulation on the 2000 population census of Jinan city (2002)
S ratio	the ratio of new construction area with S structure	%	5	Tabulation on the 2000 population census of Jinan city (2002)
BC Stock	the residential building stock with BC structure	Million m^2^	436.7	Jinan statistic yearbook (1984)
SC stock	the residential building stock with SC structure	Million m^2^	126.46	Jinan statistic yearbook (1984)
BW stock	the residential building stock with BW structure	Million m^2^	62.57	Jinan statistic yearbook (1984)
Total residential stock	the total residential building stock	Million m^2^	625.73	Jinan statistic yearbook (1984)
Refurbishment rate	the rate of refurbishment residential building per year	%	80	Survey data of China Architecture Design & Research Group (2014)
Recycling rate of steel scrap	the rate of steel scrap by recycling per year	%	0	Survey data from Construction and Demolition sites
Recycling rate of brick and concrete waste	the rate of brick and concrete waste by recycling per year	%	0	Survey data from Construction and Demolition sites
Recycling rate of glass waste	the rate of glass waste by recycling per year	%	0	Survey data from Construction and Demolition sites
Combustible rate	the rate of combustible C&D waste disposal by incineration	%	0	Survey data from Construction and Demolition sites

**Table 2:** The lifetime of residential buildings with different building structures.

	Design Lifetime/Year	Actual Lifetime/Year
BW	40	30
BC	50	30
SC	70	40
S	80	50

**Table 3:** Environmental and resource impacts’ coefficients of some variables.

	Coefficients	Unit	Value	Source
Environmental pollution	Leachate	m^3/^t	0.15	Wang et al., 2007
	Land space occupation	m^2^/10^4^ t	0.06	Yuan et al., 2006
GHG emission	C&D waste disposal by landfill	t CO_2_eq /t	0.73316	Fang, 2016
	C&D waste for incineration	t CO_2_eq/t	0.002	Fang, 2016
	Steel production	t CO_2_eq/t	1.96	Ecoinvent
	Cement production	t CO_2_eq/t	0.892	
	Wood production	t CO_2_eq/t	0.024	
	Brick and Tile production	t CO_2_eq/t	0.0521	
	Sand production	t CO_2_eq/t	0.0119	
	Stone production	t CO_2_eq/t	0.0663	
	Glass production	t CO_2_eq/t	0.731	
	Linoleum production	t CO_2_eq/t	1.06	
	Pitch production	t CO_2_eq/t	0.459	

**Table 4:** Comparison between the simulated data and actual data.

Year	Urban Population/million		Urban Residential Building Stock/million m^2^	
	Actual Data	Simulation Data	Actual Data	Simulation Data
1978	1.94	1.77	7.87	6.35
1979	1.88	1.84	7.94	7.31
1980	1.9	1.92	8	8.05
1981	1.92	2	8.45	8.74
1982	1.98	2.07	9.03	9.43
1983	2.03	2.15	10.03	10.13
1984	2.08	2.23	10.58	10.86
1985	2.11	2.3	11.01	13.06
1986	2.15	2.38	15.92	16
1987	2.19	2.45	16	17.4
1988	2.23	2.52	16.74	18.32
1989	2.27	2.59	17.02	19.08
1990	2.56	2.66	19.34	19.79
1991	2.85	2.73	21.65	20.49
1992	2.87	2.8	21.98	21.17
1993	2.9	2.86	22.61	21.85
1994	2.93	2.92	23.14	22.51
1995	2.97	2.98	23.75	23.17
1996	3.01	3.04	24.08	24.26
1997	3.05	3.09	24.7	26.04
1998	3.08	3.15	30.5	28.05
1999	3.12	3.2	31.15	30.16
2000	3.15	3.25	33.09	32.33
2001	3.2	3.29	34.22	40.93
2002	3.25	3.34	57.95	53.16
2003	3.31	3.38	62.43	59.23
2004	3.38	3.42	65.96	63.18
2005	3.45	3.46	67.41	66.37
2006	3.5	3.49	70.37	69.3
2007	3.53	3.53	74.03	72.11
2008	3.51	3.56	75.57	81.38
2009	3.49	3.59	102.68	96.78
2010	3.48	3.62	103.39	108.94
2011	3.49	3.64	105.67	119.79
2012	3.51	3.67	119.28	130.08
2013	3.54	3.69	133.29	140.13
2014	3.58	3.72	148.11	150.06
2015	3.63	3.74	163.25	158.12
	Error	2.76%	Error	1.44%

**Table 5:** The proposed policy measures.

Policy Measures Implication		Description
**Single policy**	Policy 1: compact PCFA policy	The PCFA will be restricted 45 m^2^ from 2016 to 2100.
	Policy 2: new technology policy	65% of the new residential building will be building with steel structure by 2050.
	Policy 3: long lifetime policy	The lifetime of BC and SC and S building will be extended to 40 years, 50 years, and 60 years, respectively, from 2016 to 2100.
	Policy 4: high recycle rate policy	90% of steel scraps, 70% of the cement and brick waste and 80% of the glass waste from C&D waste will be recycled respectively by 2050. 90% of the combustible C&D waste will be incinerated by 2050.
**Complex policy**	Policy 1+2	Policy 1+Policy 2
	Policy 1+3	Policy 1+Policy 3
	Policy 1+4	Policy 1+Policy 4
	Policy 2+3	Policy 2+Policy 3
	Policy 2+4	Policy 2+Policy 4
	Policy 3+4	Policy 3+Policy 4
	Policy 1+2+3	Policy 1+Policy 2+Policy 3
	Policy 1+2+4	Policy 1+Policy 2+Policy 4
	Policy 1+3+4	Policy 1+Policy 3+Policy 4
	Policy 2+3+4	Policy 2+Policy 3+Policy 4
	Policy 1+2+3+4	Policy 1+Policy 2+Policy 3+Policy 4

## Data Availability

All data generated or analyzed during this study are included in this published article.
